# Supplementary figures and images for: The quorum sensing transcription factor AphA directly regulates natural competence in Vibrio cholerae
Source: PLoS Genet. 2019 Oct 28;15(10):e1008362. doi: 10.1371/journal.pgen.1008362 (PMC6855506; doi:10.1371/journal.pgen.1008362)

**Figure S1**

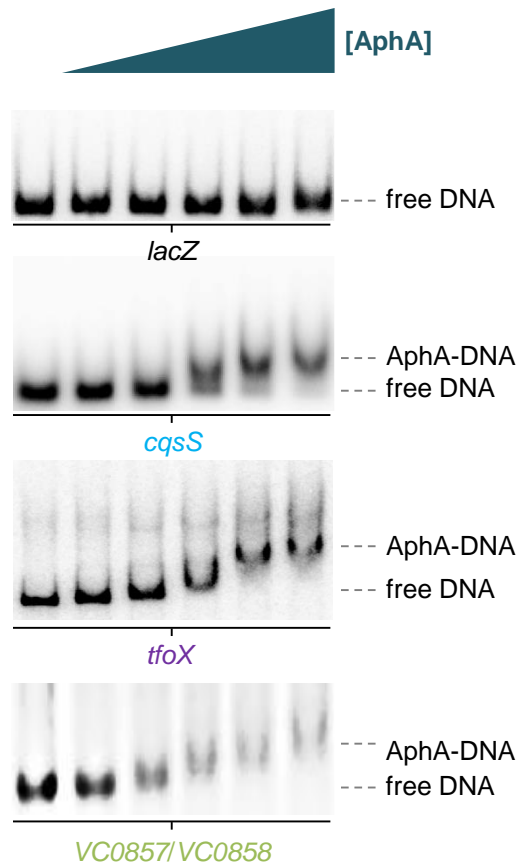

Supplement: S1 Fig — The gel images show results of electrophoretic mobility shift assay using DNA fragments corresponding to the indicated intergenic DNA regions. The lacZ promoter region used was from E. coli whilst other DNA fragments were derived from V. cholerae. (PDF) [file pgen.1008362.s001.pdf]

Figure S2

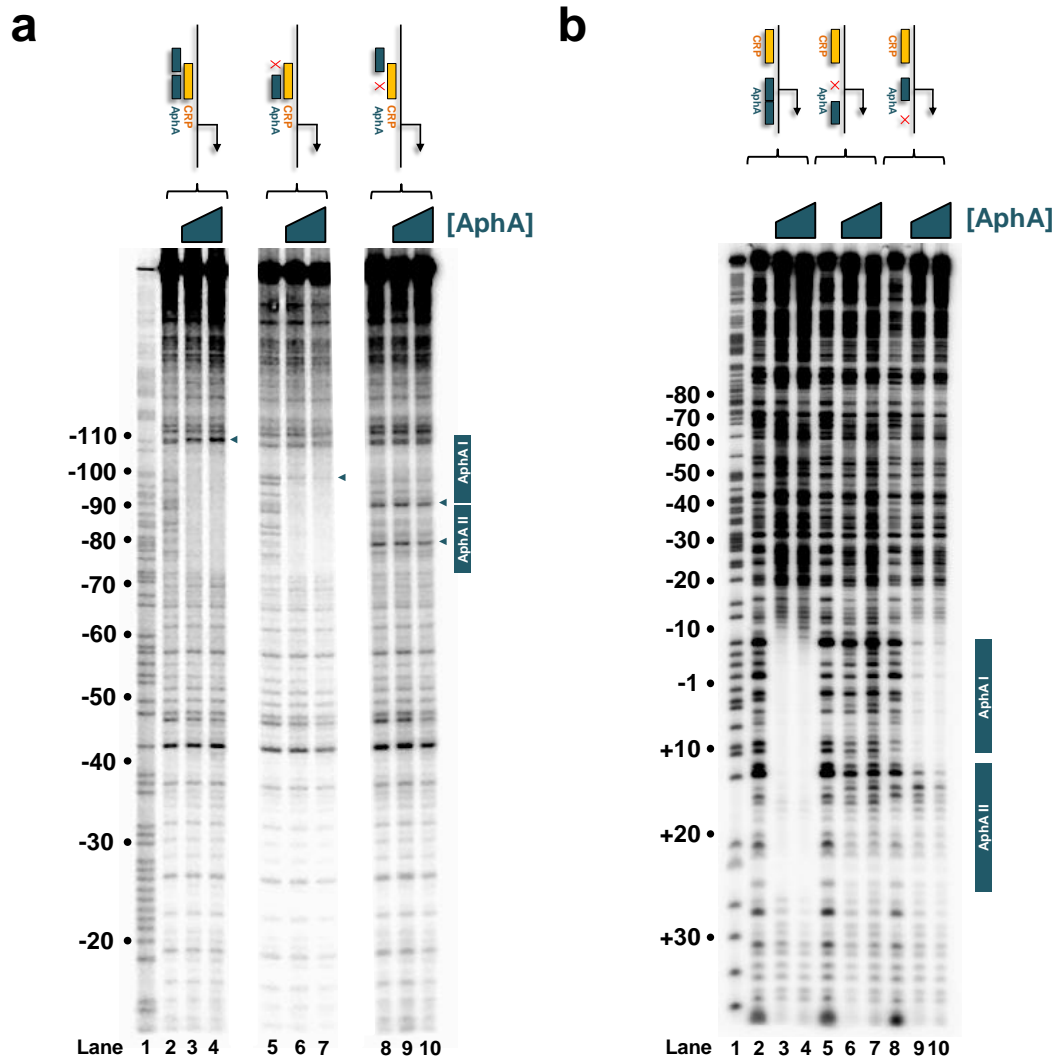

Supplement: S2 Fig — a. Binding of AphA to the tfoX regulatory region and derivatives. The gel shows the result of a DNase I footprint using DNA fragments containing the tfoX regulatory region or derivatives with point mutations in individual AphA sites. Lanes 1–4 show results for the wild type DNA fragment. Lanes 5–7 and 8–10 show results with AphA I or AphA II mutated respectively. A Maxim-Gilbert ‘G+A’ ladder has been used to calibrate the gel (lane 1). Lanes 2, 5 and 8 show the pattern of DNase I cleavage in the absence of AphA. Where added, AphA was present at concentrations of 0.5 or 1.0 μM. b. Binding of AphA to the cqsS regulatory region and derivatives. The gel shows the result of a DNase I footprint using DNA fragments containing the cqsS regulatory region or derivatives with point mutations in individual AphA sites. Lanes 1–4 show results for the wild type DNA fragment. Lanes 5–7 and 8–10 show results with AphA I or AphA II mutated respectively. A Maxim-Gilbert ‘G+A’ ladder has been used to calibrate the gel (lane 1). Lanes 2, 5 and 8 show the pattern of DNase I cleavage in the absence of AphA. Where added, AphA was present at concentrations of 2.0 or 3.0 μM. (PDF) [file pgen.1008362.s002.pdf]

**Figure S3**

**a**

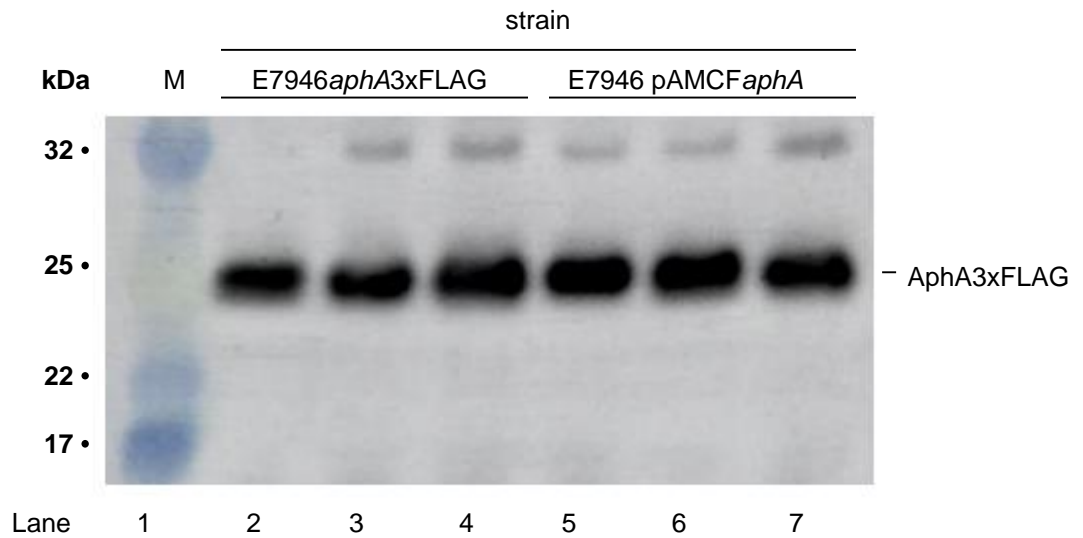

**b**

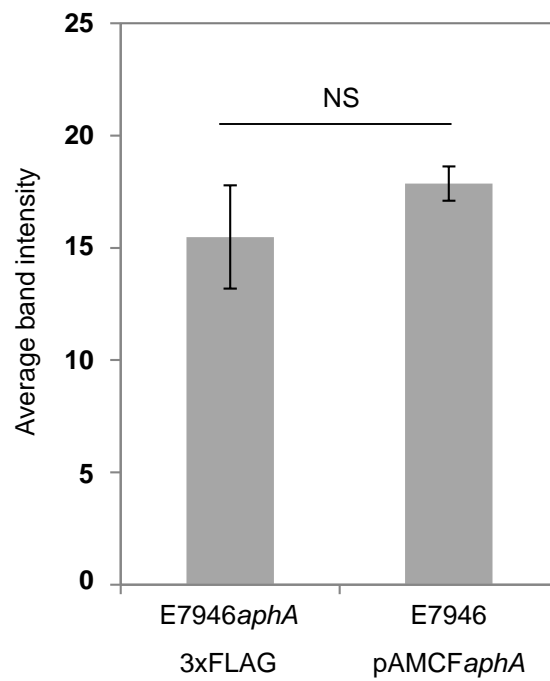

Supplement: S3 Fig — a. Image of a western blot to compare levels of AphA3xFLAG generated from the native chromosomal locus for aphA (lanes 2–4) and from plasmid pAMCFaphA (lanes 5–7). Individual lanes are replicates and equal amounts of total cellular protein were loaded in each lane. Cultures were harvested at an OD650 of 0.6. b. Quantification of relative AphA3xFLAG levels. The AphA3xFLAG band intensity for each lane in panel a was determined using Quantity One software. The average band intensity is shown and error bars represent the standard deviation of the three experimental replicates shown in panel a. P = 0.299 was calculated using a two-tailed Student’s t-test and there was no significant difference in AphA levels when comparing the two expression methods (NS). (PDF) [file pgen.1008362.s003.pdf]

**Figure S4**

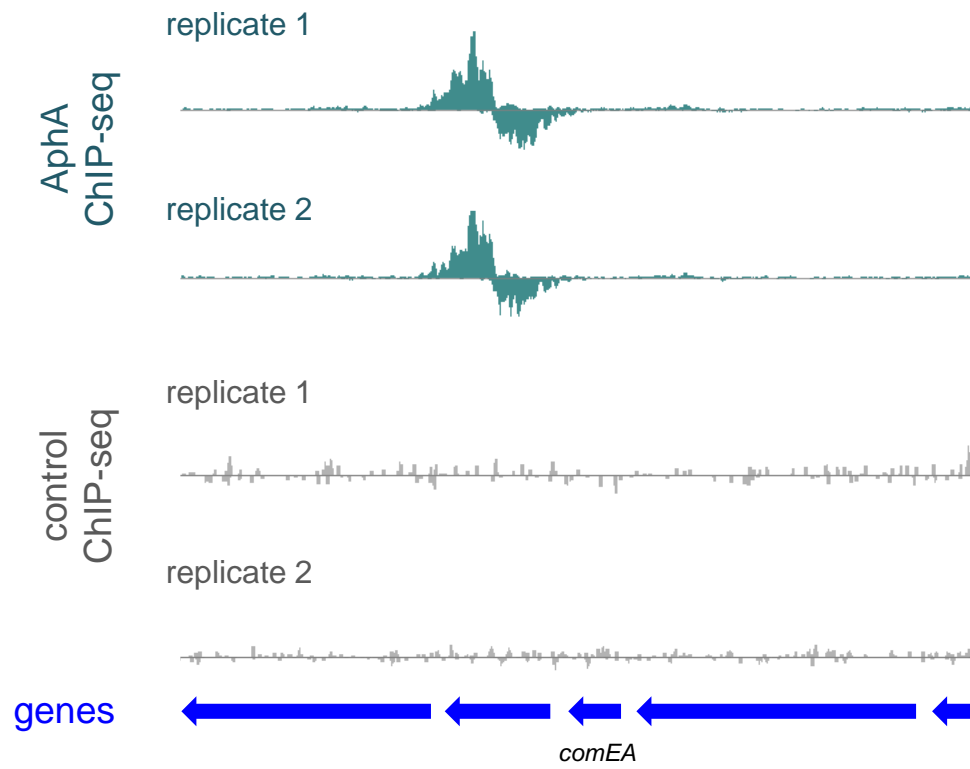

Supplement: S4 Fig — Genes are shown as block arrows. ChIP-seq coverage plots are shown for individual experimental replicates. Data for AphA are in teal and control profiles are grey. Signals above or below the horizontal line correspond to reads mapping to the top or bottom strand respectively. (PDF) [file pgen.1008362.s004.pdf]

**a**

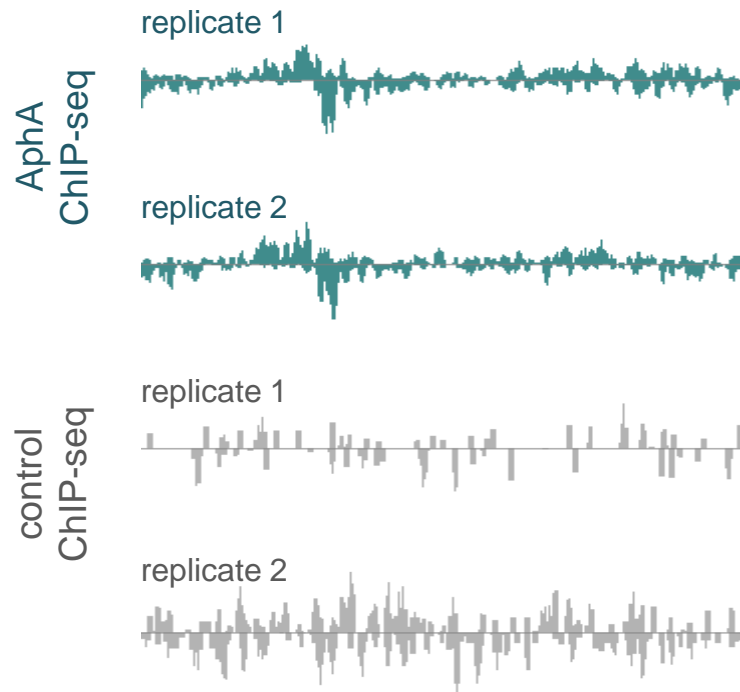

**b**

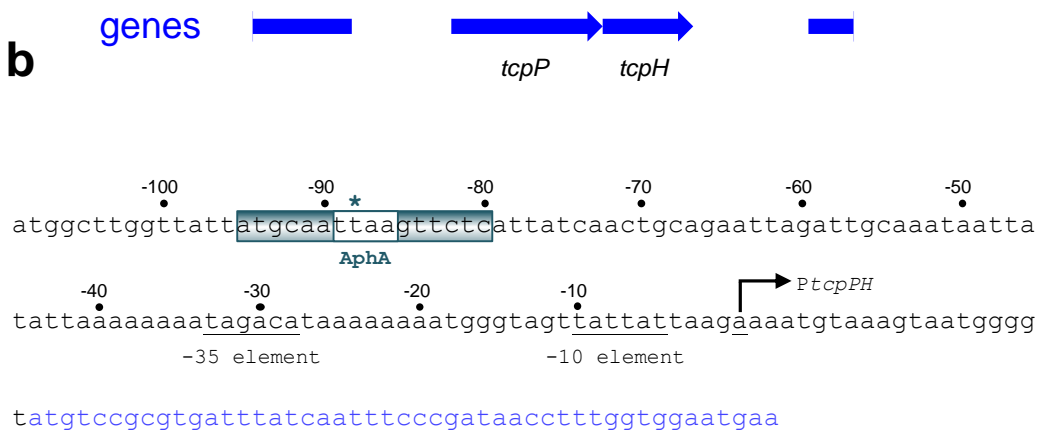

Supplement: S5 Fig — a. ChIP-seq data for AphA binding at the tcpPH locus. Genes are shown as block arrows. ChIP-seq coverage plots are shown for individual experimental replicates. Data for AphA are in teal and control profiles are grey. Signals above or below the horizontal line correspond to reads mapping to the top or bottom strand respectively. b. Sequence of the tcpPH intergenic region. Coding DNA is in blue. Promoter elements are underlined and labelled. The transcription start site is shown as a bent arrow. Distances are with respect to the tcpPH transcription start site. The centre of the AphA ChIP-seq peak is denoted by a teal asterisk. The known AphA binding site is boxed. (PDF) [file pgen.1008362.s005.pdf]

# Figure S6

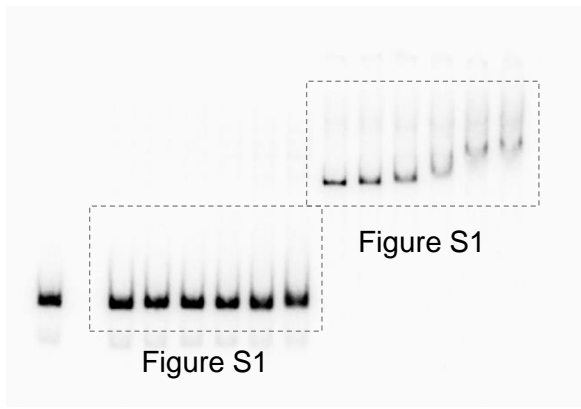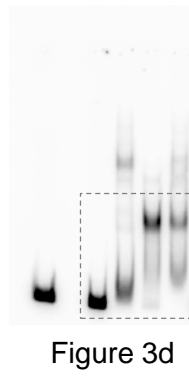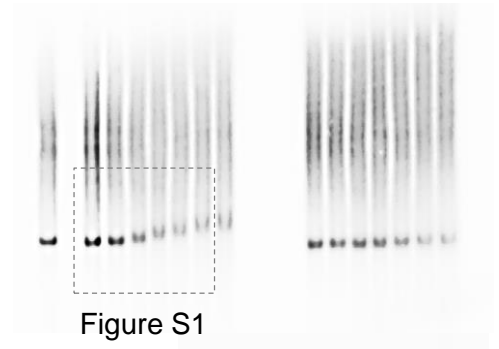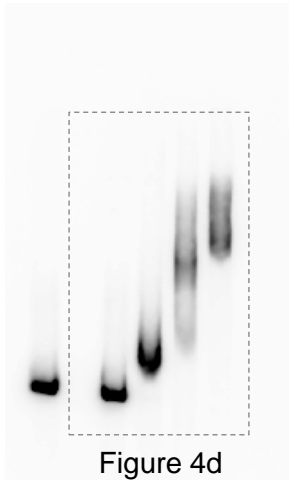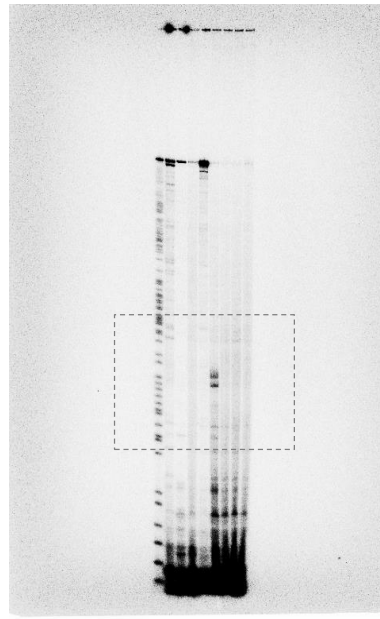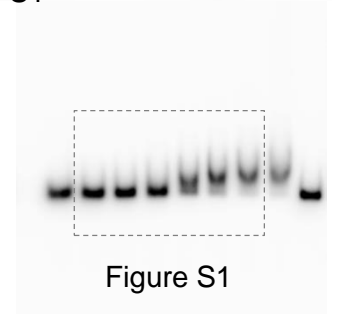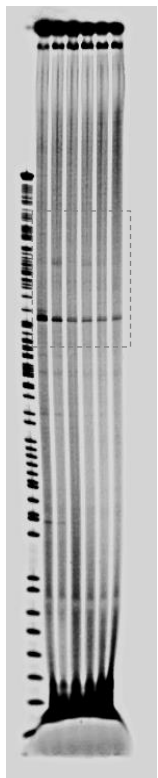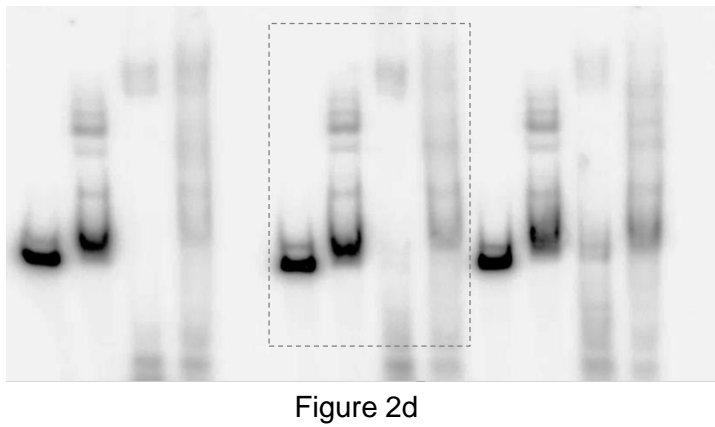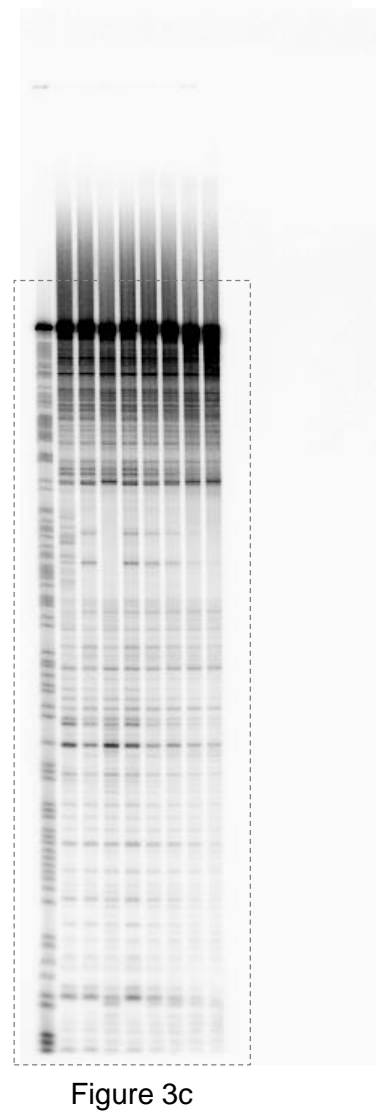

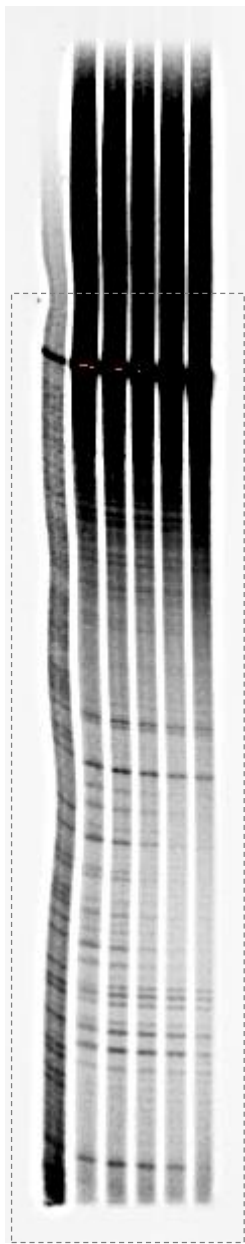

Figure 2c

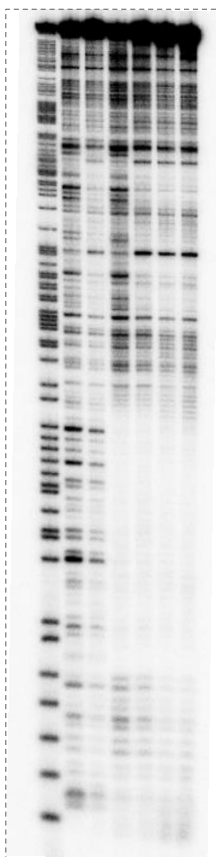

Figure 4c

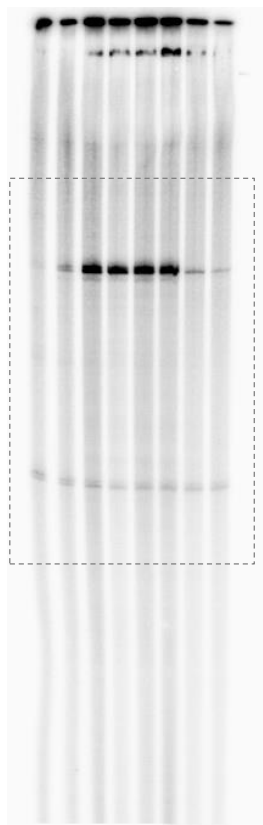

Figure 3e

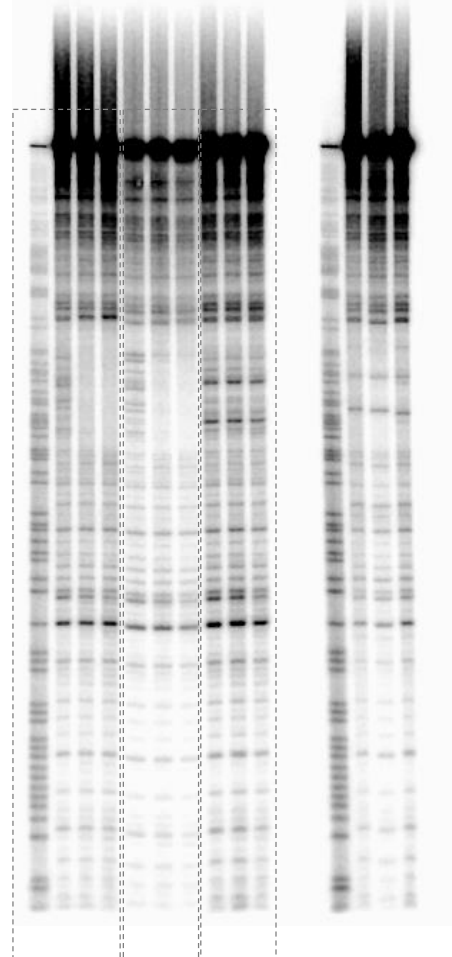

Figure S2a

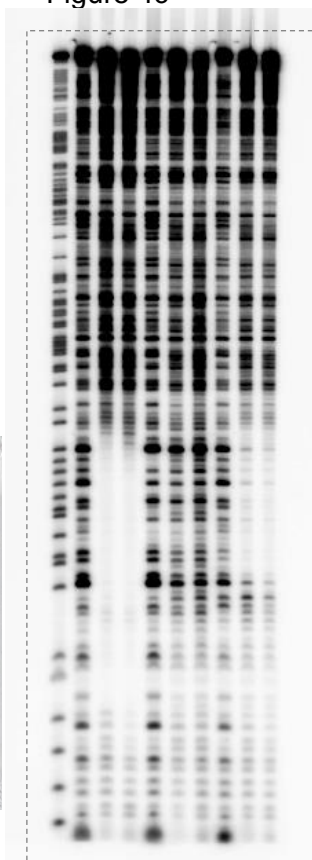

Figure S2b

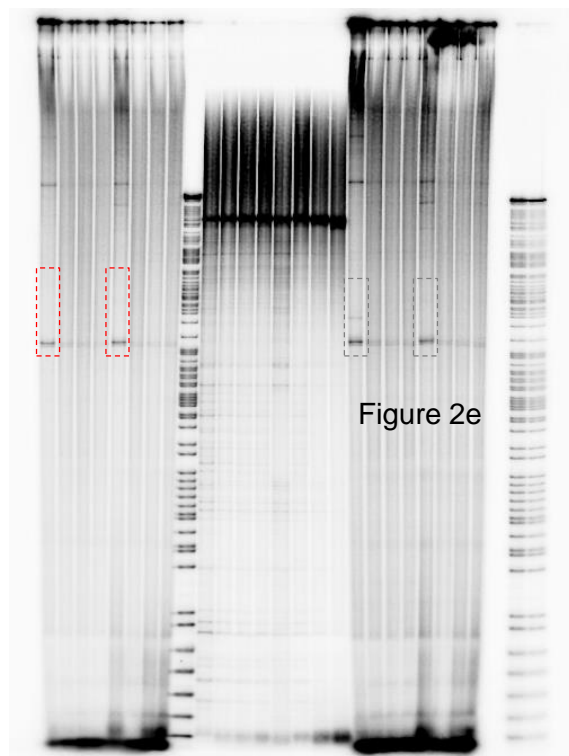

Figure 2e

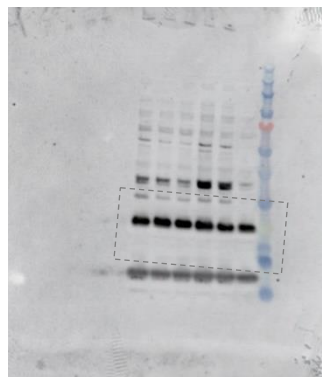

Figure S5

Supplement: S6 Fig — Complete raw gel images are shown and subsections of images used for figures are indicated. (PDF) [file pgen.1008362.s006.pdf]
