# Supplementary material for: The quorum sensing transcription factor AphA directly regulates natural competence in Vibrio cholerae
Source: PLoS Genet. 2019 Oct 28;15(10):e1008362. doi: 10.1371/journal.pgen.1008362 (PMC6855506; doi:10.1371/journal.pgen.1008362)
Supplement: S1 Table — (DOCX) [file pgen.1008362.s009.docx]

**Table S1: Strains, plasmids and oligonucleotides**

**Name Description Source**

**Strains**

*Vibrio cholerae* E7946 SmR Wildtype *Vibrio cholerae*, El Tor Ogawa derivative, SmR Levine *et al*., 1982

*Vibrio cholerae* E7946 Δ*aphA* E7946 derivative lacking the *aphA* gene This study

SAD2413 E7946 SmR derivative, ∆*lacZ*::*lacIq*, Ptac-*tfoX*, *pilA* S67C, This study

∆VC1807::CmR, pAMNF*aphA* KanR

SAD2414 E7946 SmR derivative, ∆*lacZ*::*lacIq*, Ptac-*tfoX*, *pilA* S67C, This study

∆VC1807::CmR, pAMCF*aphA* KanR

SAD2415 E7946 SmR derivative, ∆*lacZ*::*lacIq*, Ptac-*tfoX*, ∆*luxO*, *pilA* S67C This study

∆VC1807::ZeoR pAMNF*aphA* KanR

SAD2416 E7946 SmR derivative, ∆*lacZ*::*lacIq*, Ptac-*tfoX*, ∆*luxO*, *pilA* S67C This study

∆VC1807::ZeoR pAMCF*aphA* KanR

SAD2424 E7946 SmR derivative, ∆*lacZ*::*lacIq*, Ptac-*tfoX*, ∆*luxO*, comEA-mCherry, This study

*pilA* S67C ∆VC1807::CmR, pAMNF*aphA* KanR

SAD2425 E7946 SmR derivative, ∆*lacZ*::*lacIq*, Ptac-*tfoX*, ∆*luxO*, comEA-mCherry, This study

*pilA* S67C ∆VC1807::CmR, pAMCF*aphA* KanR

SAD2062 E7946 SmR derivative, ∆VC1807::CmR, *luxO* D61E This study

SAD2063 E7946 SmR derivative, ∆VC1807::CmR, *luxO* D61E, ∆*aphA* This study

JLC937 E7946 SmR derivative, ∆*dns*::SpecR, *luxO* D61E This study

JLC938 E7946 SmR derivative, ∆*dns*::SpecR, *luxO* D61E, ∆*aphA* This study

*E. coli* DH5α F–*endA*1, *gln*V44, *thi*-1, *recA1*, *relA1*, *gyrA96*, *deoR*, *nupG*, *purB20*, Taylor *et al*., 1993

φ80d*lac*ZΔM15, Δ(*lacZYA-argF*) U169, *hsdR17*

*E. coli* DH5α (pRK2013) DH5α, KmR, oriColE1, RK2- Mob+ Tra+ Connell *et al*., 1995

*E. coli* JCB387 *ΔnirB* *Δlac* Page *et al*. 1990

T7 express *fhuA2* *lacZ*::T7 gene1 [lon] *ompT* *gal* *sulA11* R(*mcr73*::miniTn10--TetS)2 Invitrogen

[*dcm*] R(*zgb*-210::Tn10--TetS) *endA1* ∆*mcrCmrr*)114::IS10

**Plasmids**

pAMNF pJ201-derived plasmid encoding 3x FLAG upstream of a *Kpn*I-*Hin*dIII cloning Sharma *et al*., 2017

site. Expression of fusion proteins drive by a low-level constitutive promoter.

pUC origin.

pAMCF pJ201-derived plasmid encoding 3x FLAG downstream of a *Kpn*I-*Hin*dIII Sharma *et al*., 2017

cloning site. Expression of fusion proteins drive by a low-level constitutive

promoter. pUC origin. KmR.

pDCRP-*Vc* Encodes *crp* under the control of its native promoter (located on *Eco*RI-*Sal*I Manneh-Roussel *et al*.,

flanked fragment). pBR322 derived. ApR. ColE1 origin. 2018

pRW50T pRW50-derived broad-host-range lac fusion vector for cloning promoters on Manneh-Roussel *et al*.

*Eco*RI–*Hin*dIII fragments: contains the RK2 origin of replication and encodes 2018

TcR.

pSR pBR322-derived plasmid containing an *Eco*RI–*Hin*dIII cloning site upstream of Kolb *et al.,* 1995

the λ*oop* transcription terminator. ApR.

pMMB-*tfoX* pMMB67EH-derived plasmid encoding *tfoX* under the control of an Hayes *et al.,* 2017

IPTG-inducible promoter.

**Oligonucleotides**

*Oligonucleotides used to clone* aphA *into pAM plasmids (5'-3')*

*aphA* N-ter forward ggctgcggtacctcattaccacacgttatccttactgttcttagc This study

*aphA* N-ter reverse gcccgaagcttttatgccatcgcgttcaattctgccaac This study

*aphA* C-ter forward ggctgcggtaccatgtcattaccacacgttatccttactgttc This study

*aphA* C-ter reverse gcccgaagctttgccatcgcgttcaattctgccaac This study

*Oligonucleotides used for strain construction (5'-3')*

Mugent Δ*aphA*_left arm F1 cggattactgcatcatcggtgaacc This study

Mugent Δ*aphA*_left arm R2 gtttgcccactcgaagaggagatgc This study

Mugent Δ*aphA*_right arm F2 tttacatcgacaggtttggcttggcgtcttcaatccaaaaattaagtcaa This study

Mugent Δ*aphA* right arm R1 ttgacttaatttttggattgaagacgccaagccaaacctgtcgatgtaaa This study

Δ*aphA* MASC forward ggctttataaggtgacataagcag This study

Δ*aphA* MASC reverse ctctcacggtatttaagtactcattcc This study

∆*dns*_left arm F1 gatggttttggtacaaatcagcg This study

∆*dns*_left arm R1 gtcgacggatccccggaatcatcataaaagacgtagataagtagg This study

∆*dns*_right arm F2 gaagcagctccagcctacaaactgatgggcagaatctcac This study

∆*dns*_right arm R2 atcgctactatgtgttgcaacg This study

∆VC1807_left arm F1 tttaaaggggatcagtgaccg This study

∆VC1807_left arm R1 gtcgacggatccccggaatacgtttcattagtcacctctattgttaacttgttc This study

∆VC1807_right arm F1 gaagcagctccagcctacatagtcgaaaataaaaaaaagaggctcgcctc This study

∆VC1807_right arm R2 caattttgcttttggaccatccc This study

SpecR CmR ZeoR cassettes F attccggggatccgtcgac This study

SpecR CmR ZeoR cassettes R tgtaggctggagctgcttc This study

*luxO* D61E_left arm F1 tggcaaaaagcgagagaagaag This study

*luxO* D61E_left arm R1 tcatatctggcaaacgtaactccagcaggattaagtcagg This study

*luxO* D61E_right arm F2 tgacttaatcctgctggagttacgtttgccagatatgacg This study

*luxO* D61E_right arm R2 tcacacccgaatttccatcatgc This study

*luxO* D61E detect F atcgttcttacctcacaccg This study

*luxO* D61E detect R tcatatctggcaaacgtagc This study

*Oligonucleotides for cloning* aphA *into pET21a (5'-3')*

*aphA* forward tatgatcatatgtcattaccacacgttatccttactgttc This study

*aphA* reverse ctcatactcgaggctgccgcgcggcaccagtgccatcgcgttcaattctgccaac This study

*Oligonucleotides for sequencing pSR and pRW50T constructs (5'-3')*

pSR forward gcatttatcagggttattgtctc This study

pSR reverse catcaccgaaacgcgcgagg This study

pRW50 forward gttctcgcaaggacgagaatttc This study

pRW50 reverse aatcttcacgcttgagatac This study

*Oligonucleotides for generating PVC0858- PVC0858 derivatives (5'-3')*

VC0857 forward: ggctgcgaattccatttcccaaaatcctttgcaaaccgtgagaggatgatgaatcaacttgcatagatgc This study

VC0857 reverse cgcccgaagcttcatttccattacttctctttataaaacaacgcc This study

VC0857 AphA site mutated ggctgcgaattccatttcccaaaatcctttgcaaaccgtgagggcgtgcaacttcgatagatgctaaca This study

forward gctatc

VC0858 forward ggctgcgaattccatttccattacttctctttataaaacaacgcc This study

VC0858 reverse cgcccgaagcttcatttcccaaaatcctttgcaaaccgtgagaggatgatgaatcaacttgcatagatgc This study

VC0858 AphA site mutated cgcccgaagcttcatttcccaaaatcctttgcaaaccgtgagggcgtgcaacttcgatagatgctaacag This study

reverse ctatc

*Oligonucleotides for generating P*tfoX *derivatives (5'-3')*

P*tfoX* forward ggctgcgaattctagaggagcaacaaaaaattgacgctgtg This study

P*tfoX* reverse cgcccgaagcttgcttgttttatgaagctaatgatagtttacgaagag This study

P*tfoX* AphA I mutated ggctgcgaattctagaggagcaagaaaaaatacacgctgtgatactggtc This study

forward

P*tfoX* AphA II mutated ggctgcgaattctagaggagcaacaaaaaattgacgctgtgactctggtcatataatctggcgcaa This study

forward

P*tfoX* long forward (used ggctgcgaattccataaaatacacgatcaagcaaagttggtagc This study

for cloning into pSR and

pRW50T for *in vitro* transcription

and LacZ assays)

P*tfoX* long reverse (used for cgcccgaagcttcattcatatccattgatcctttaatcacgttcccc This study

cloning into pSR and

pRW50T *in* *vitro*

Transcription and LacZ assays)

*Oligonucleotides for generating P*cqsS *derivatives (5'-3')*

P*cqsS* forward ggctgcgaattcggtagcacgctgcgtttaatcacataag This study

P*cqsS* reverse cgcccgaagcttcatgctcactatcactaccgttgc This study

P*cqsS* AphA I mutated cgcccgaagcttcatgctcactatcactaccgttgcattctcttgctaaacttcaacacgaacatacttta This study

reverse ccgtttcgc

P*cqsS* AphA II mutated cgcccgaagcttcatgctcactatcactaccgttgcgttctcttagtaatcatcaacatgcacatacttta This study

reverse ccgtttcgc

*Oligonucleotides used for amplifying Ab^R^ for transformation assays (5'-3')*

BBC1881  tttaaaggggatcagtgaccg This study BBC1882 caattttgcttttggaccatccc This study

**REFERENCES**

Connell TD, Martone AJ, Holmes RK. 1995. A new mobilizable cosmid vector for use in *Vibrio cholerae* and other gram-negative bacteria. *Gene*. **153:**85-87.

Hayes, C. A., Dalia, T. N. and Dalia, A. B. 2017. Systematic genetic dissection of chitin degradation and uptake in *Vibrio* *cholerae*. *Environ* *Microbiol.* **19**: 4154-4163.

Kolb A, Kotlarz D, Kusano S, Ishihama A. 1995. Selectivity of the *Escherichia coli* RNA polymerase Eσ38 for overlapping promoters and ability to support CRP activation. *Nucleic Acids Res*. **23:**819-826.

Levine MM, Black RE, Clements ML, Cisneros L, Saah A, Nalin DR, Gill DM, Craig JP, Young CR, Ristaino P. 1982. The pathogenicity of nonenterotoxigenic *Vibrio cholerae* serogroup O1 biotype El Tor isolated from sewage water in Brazil*. J Infect Dis*. **145:**296-299

Manneh-Roussel J, Haycocks JRJ, Magán A, Perez-Soto N, Voelz K, Camilli A, Krachler AM, Grainger DC. 2018. cAMP Receptor Protein Controls *Vibrio cholerae* Gene Expression in Response to Host Colonization. *mBio*. **9:**e00966-18.

Page L, Griffiths L, Cole JA. 1990. Different physiological roles of two independent pathways for nitrite reduction to ammonia by enteric bacteria. *Arch Microbiol*. **154:**349-354.

Sharma P, Haycocks JRJ, Middlemiss AD, Kettles RA, Sellars LE, Ricci V, Piddock LJV, Grainger DC. 2017. The multiple antibiotic resistance operon of enteric bacteria controls DNA repair and outer membrane integrity. *Nat Commun*. **8:**1444.

Taylor RG, Walker DC, McInnes RR. 1993. *E. coli* host strains significantly affect the quality of small scale plasmid DNA preparations used for sequencing. *Nucleic Acids Res*. **21:**1677-1678.
